# Protective cellular immunity generated by cross-presenting recombinant overlapping peptide proteins

**DOI:** 10.18632/oncotarget.20407

**Published:** 2017-08-24

**Authors:** Lili Cai, Jianbo Zhang, Renying Zhu, Weixing Shi, Xiaobing Xia, Mark Edwards, William Finch, Anthony Coombs, Ju Gao, Kangwen Chen, Sophie Owen, Shisong Jiang, Wenshu Lu

**Affiliations:** ^1^ Oxford Vacmedix (Changzhou) Company Ltd, Changzhou, Jiangsu, China; ^2^ The No.2 People's Hospital of Dali, Dali, Yunnan, China; ^3^ Oxford Vacmedix UK Limited, Oxford, United Kingdom; ^4^ Shanghai JW Inflinhix Co Ltd, Shanghai, China; ^5^ Department of Oncology, University of Oxford, Oxford, United Kingdom

**Keywords:** vaccines, peptide immunization, overlapping peptide, antigen processing, cross-presentation

## Abstract

Priming of naive CD8^+^ and CD4^+^ T cells by dendritic cells (DCs) requires effective antigen presentation on both MHC class I and II molecules. We have developed a novel technology to use recombinant overlapping peptides (ROP) that stimulate both CD8^+^ and CD4^+^ T cell immune responses. The single chain protein of a ROP is made up of overlapping peptides linked by the target sequence (LRMK) for cathepsin S, a protease found in the endosomes of DCs. We designed synthetic genes encoding ROPs derived from ovalbumin (OVA), tuberculosis protein (CFP10-ESAT6), human papilloma virus (HPV) protein (E7) and survivin, a protein commonly over-expressed in tumour cells. An epitope from ROP-OVA was cross-presented and detected by a CD8^+^ T cell receptor-like antibody (TCR like Ab). Human DCs pulsed with ROP-survivin activated CD8^+^ T cells. CD4-low PBMCs from HIV and TB co-infected patients recognized ROP-CFP10-ESAT6 compared to a soluble form of the antigen. Immunization of mice with ROP-survivin or ROP-HPV-E7 generated specific cellular immune responses and protected mice from inoculation with melanoma B16 cells expressing survivin or HPV-E7 proteins. Together these data provide evidence to support ROP as a central component of a new platform for therapeutic vaccines and diagnostics.

## INTRODUCTION

Priming naive antigen-specific CD8^+^ and CD4^+^ T cells requires effective antigen presentation by MHC class I and II molecules on the surface of antigen presenting cells (APC), such as dendritic cells (DC) [1, 2]. For CD8^+^ T cells, an antigen must be endogenous, i.e. expressed in or delivered to the cytoplasmic compartment of an APC, before being processed to produce peptide epitopes that are bound to MHC class I in the endoplasmic reticulum (ER) [3]. In contrast, exogenous antigens are endocytosed by APCs into endosomes before merging with lysosomes. Here the antigens are degraded and processed, resulting in peptide epitopes bound to MHC class II. The MHC-peptide complex then moves to the surface of the APC where it can stimulate CD4^+^ T cells [4]. In some situations, cross-presentation, a process in which exogenous antigen is presented by MHC class I on APCs to activate CD8^+^ T cells occurs. The mechanisms by which this cross-presentation occurs remain unclear [5, 6].

Cross-presentation is important for the priming of CD8^+^ T cells; however, the efficiency of this route of antigen presentation is low [6–8]. As a result, current strategies for stimulating CD8^+^ T cell immunity are focused on *in situ* expression of antigens, achieved by delivering DNA encoding the target protein into the cytoplasm of APCs. This is the basis of DNA vaccines or immunization using bacterial or viral vectors encoding target antigens as vaccines. These vaccines have been in development for a number of years, but so far most of them remain in experimental stages. One limitation they share is that they are associated with unwanted immune responses to vector elements that can suppress immunity to the target antigen [9].

Along with others, we have demonstrated that an exogenously applied pool of overlapping peptides is able to stimulate both CD4^+^ and CD8^+^ T cell immunity to a level that has clinical significance [10–12]. Moreover, we have demonstrated that a pool of overlapping peptides is more effective than the native protein in antigen presentation [13, 14]. Furthermore, the use of overlapping peptides more comprehensively represents the range of potential T cell epitopes. With a view to reducing the cost of manufacture and overcoming the regulation difficulties of multiple synthetic peptides, we made an artificial protein composed of overlapping peptides taken from the target protein, interspersed by a protease cleavage sequence. We describe such artificial antigens as recombinant overlapping peptide proteins (ROPs). The ROP version of an antigen can be cleaved into overlapping peptides *in vitro* by incubation with the relevant protease [14]. This ROP approach greatly reduces the cost of producing a pool of peptides and has shown efficacy in stimulating both CD8^+^ and CD4^+^ immune responses and protection from viral infection in animals [14].

However, there are several caveats that may impede the development of this technology, mainly from the manufacturing and regulation point of view. First, it is difficult to quality-control the manufacture of the peptide mixtures and achieve batch to batch consistency. Secondly, similar to using overlapping synthetic peptides as vaccines, a pool of the peptides may be regarded as multiple entities when applying for an approval from a regulatory body. Thirdly, the involvement of protease *in vitro* to digest the ROP vaccines and the extra procedures to separate the enzyme from the mixture of peptides afterward may result in additional regulatory hurdles and costs. These drawbacks reduce the potential benefits of using ROPs in vaccine development.

Protein-based subunit vaccines are exogenous antigens and as such are not efficient in stimulating CD8^+^ T cell immunity. Nonetheless, vaccines of this type are widely used and have many advantages over other forms of vaccine for the purposes of manufacture, regulatory approval and vaccine administration. This led us to consider the use of intact ROPs for immunization. It occurred to us that a ROP protein could be cleaved into the desired set of overlapping peptides within the endosomal compartment, if the peptides are joined by a linker sequence recognized by an APC protease. We hypothesized that if the peptides had access the cytoplasmic compartment, they might be transported to the lumen of the ER where they would bind MHC class I molecules with subsequent presentation on the cell surface.

In this study, we adapt the synthetic gene approach previously used in ROP production to test this endogenous cleavage and cross-presentation. We explore the ability of exogenously administered artificial protein to undergo digestion within the APC and to stimulate both CD8^+^ and CD4^+^ T cells. We use ovalbumin as a model antigen to determine the ability of exogenous ROP-ovalbumin (ROP-OVA) to lead to cross-presentation of the major T cell epitope (SIINFEKL). We then explore the wider applicability of the approach by examining T cell responses induced by ROPs based on three clinically significant antigens: human papillomavirus E7, the tuberculosis protein CFP10-ESAT6 and survivin, an antigen up-regulated in a large number of tumour types.

## RESULTS

### Recombinant protein antigens containing overlapping sequences

Cathepsin S is an enzyme situated in the endosomes of an APC [14, 15]. Its main physiological function is to cleave the chaperone protein from MHC class II [15]. We therefore selected the minimal cathepsin S cleavage site (LMRK) to link the overlapping peptides in the artificial ROP vaccines [16]. To test our hypothesis, we made 4 recombinant overlapping peptides: ROP-OVA (based on ovalbumin), ROP-HPV (based on HPV E7), ROP-TB (based on CFP10 and ESAT6) and ROP-survivin.

### ROP-OVA can be cross-presented via the MHC class I pathway

**Figure 1:**
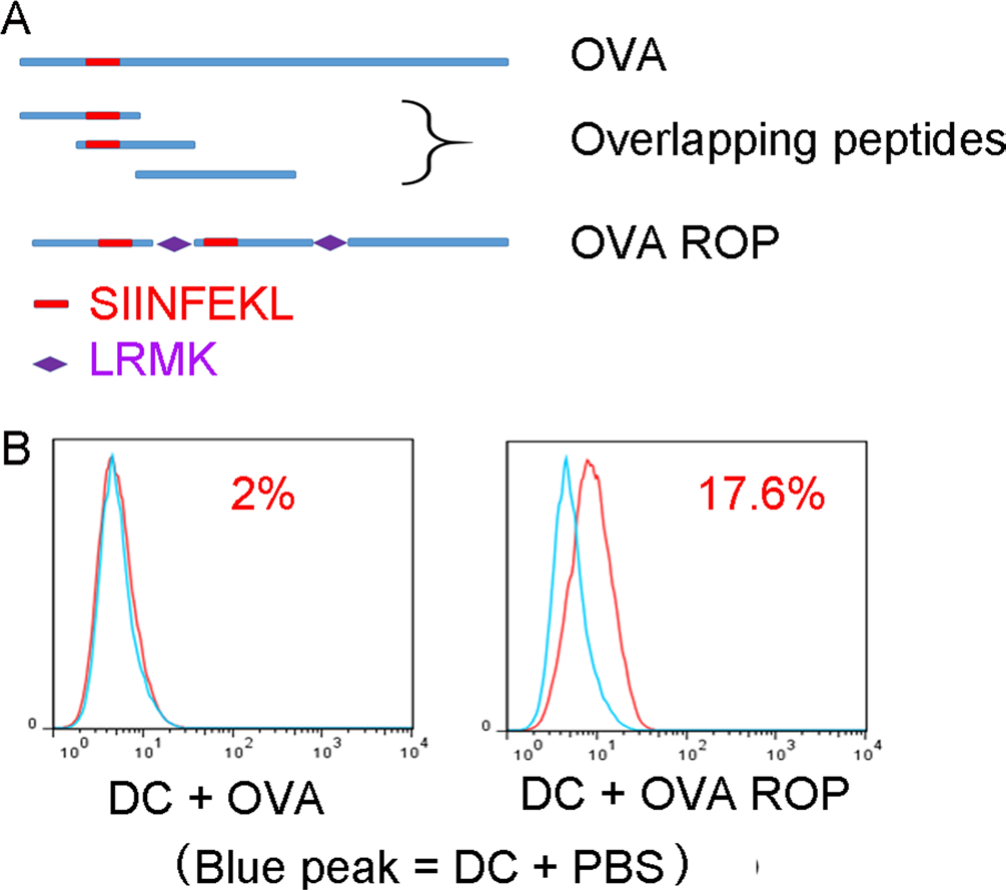
Presentation of ROP-OVA on MHC class 1 molecules (**A**) Recombinant overlapping peptides derived from native OVA were linked by LRMK, the target sequence of cathepsin S. SIINFEKL is the epitope presented on mouse H-2b. (**B**) Comparison of SIINFEKL presentation on dendritic cells after incubation of DC2.4 cells with 50 μg/ml native OVA or ROP-OVA for 13 hours. The experiment was repeated twice.

In order to assess the presentation of ROPs by MHC class I on APCs *in vitro*, we first tested the ROP-OVA construct. We examined antigen processing and presentation of ROP-OVA in DC2.4 cells (H-2b) with native ovalbumin protein (OVA) as the comparator (Figure 1A). Antigen presentation was assessed using a TCR-like antibody that specifically recognizes the ovalbumin peptide SIINFEKL when bound to the mouse MHC class I molecule H-2^b^ on the surface of APCs. No H2b-SIINFEKL complex was detected when DC2.4 cells were incubated with native OVA (Figure 1B, red peak in the left panel). However, the H-2^b^-SIINFEKL complex was readily detected after incubation of ROP-OVA with DC2.4 cells for 13 hours (Figure 1B, red peak in the right panel), indicating that ROP-OVA was efficiently cross-presented on MHC class I.

### Activation of CD8^+^ T cells by ROP-pulsed DCs

**Figure 2:**
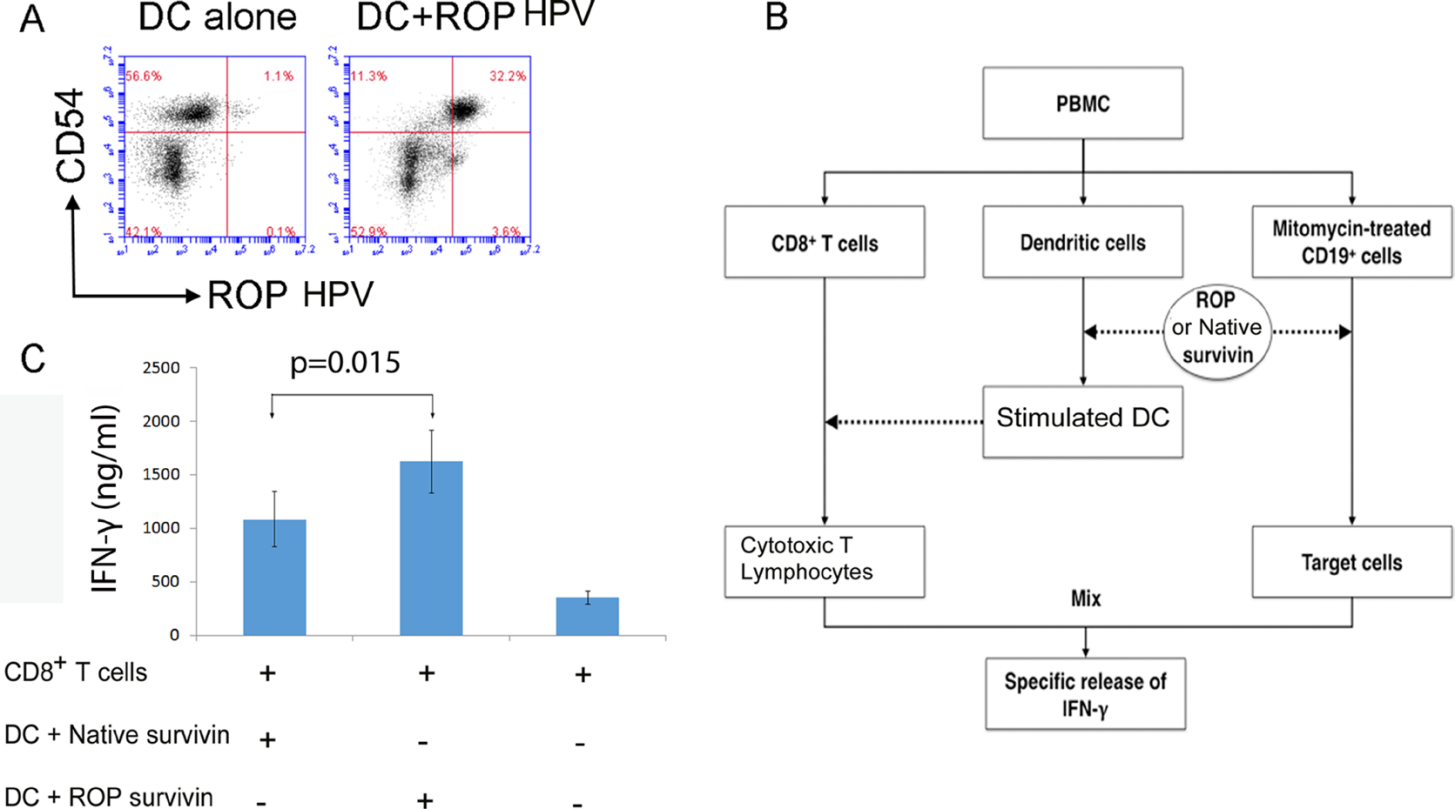
Presentation of ROP on DCs activates CD8^+^ T cells (**A**) ROP is taken up by CD54^+^ cells. PBMCs were incubated with 50 μg/ml N-HydroxySuccinimide-labelled ROP-HPV for 36 hours in the presence of 50 IU/ml IL-4 and 1000 IU/ml GM-CSF. The experiment was repeated twice. (**B**) Protocol to test for CD8^+^ killing of target cells. DCs were isolated from PBMCs and stimulated with ROP-survivin for 3 days. DCs were then cultured with naive CD8^+^ T cells for 7 days. Specific CD8^+^ T cells were measured for IFN-γ production upon culture with ROP-survivin pulsed, mitomycin treated CD19^+^ target cells. (**C**) IFN-γ production by cytotoxic T lymphocytes following incubation with ROP- survivin stimulated DCs upon culture with target cells. Naive CD8^+^ T cells following incubation with the ROP or native survivin stimulated DCs became cytotoxic T lymphocytes. ROP survivin primed CD8^+^ T cells were more cytotoxic than native Survivin primed CD8^+^ T cells. CD8^+^ T cells alone were not cytotoxic T lymphocytes, and secreted very little IFN- γ when incubated with target cells.

In order to assess the uptake of ROP antigens by human DCs, we incubated NHS (N-Hydroxy Succinimide) ester-labelled ROP-HPV with human PBMCs for 36 hours in the presence of IL-4 and GM-CSF, followed by staining for CD54. Expression of CD54 was used as a marker for an enriched population of monocyte derived DCs. Analysis by flow cytometry indicated that the majority of ROP-HPV was taken up by CD54^+^ cells (Figure 2A).

We then assessed whether ROP pulsed DCs could activate CD8^+^ T cells. CD8^+^ T cells, DCs and CD19^+^ B cells were isolated from human PBMCs. The DCs were stimulated with native survivin or ROP-survivin proteins for 3 days. CD8^+^ T cells were then co-cultured with the primed DCs cells for 7 days prior to testing them for specific killing of ROP-survivin or survivin protein pulsed target cells (mitomycin-treated autologous CD19^+^ B cells) *in vitro* (Figure 2B). Functionally, DC-ROP-survivin stimulated CD8^+^ T cells showed an increase in INF-γ secretion when incubated with ROP-bearing CD19^+^ B cells. This increased INF-γ secretion was greater for target cells primed with ROP-survivin than those primed with native survivin protein (Figure 2C, *P* = 0.015).

### ROP-TB-ESAT6-CFP10 is recognized by CD4^+^ depleted T cell populations from HIV-TB co-infected patients

To investigate whether ROP could be recognized by CD8^+^ T cells, we tested PBMCs from human immunodeficiency virus (HIV) and tuberculosis (TB) co-infected patients who had a depleted CD4^+^ T cell population but a normal CD8^+^ T cell population. We compared ROP with a current commercially available TB diagnostic kit (T-SPOT.*TB*). The kit uses two sets of overlapping synthetic peptides, T-spot 1 (overlapping peptides of ESAT6) and T-spot 2 (overlapping peptides of CFP10), to stimulate TB reactive T cells in PBMCs taken from a patient. In general, however, peptide synthesis gives lower yields and is much less cost effective than recombinant manufacture [17].

**Figure 3:**
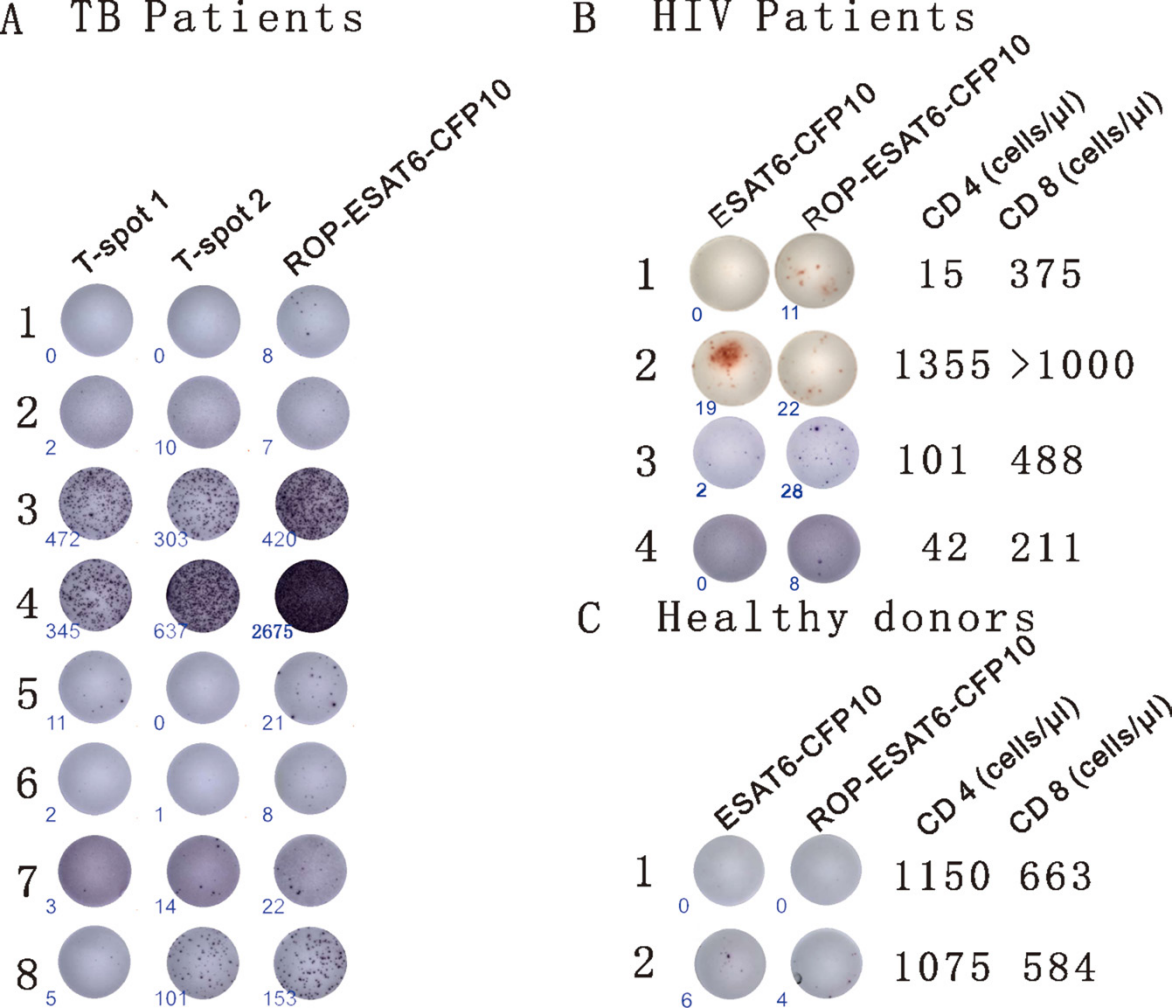
ROPs for the diagnosis of TB (**A**) Detection of TB with ROP antigens is comparable to current diagnostic tests. PBMCs from 8 TB patients (clinically diagnosed as TB infection) were incubated with the T-SPOT.TB1 (synthetic overlapping peptides of ESAT6) and T-SPOT. TB2 (synthetic overlapping peptides of CFP10) or ROP-ESAT6-CFP10 ELISpot-based tests for 13 hrs. An individual cell producing IFN-γ is represented by appearance of a spot with number of spots at lower left corner. (**B**) ROP antigen detects TB infection in HIV- related CD4^+^ depleted PBMCs. PBMCs from 4 HIV/TB co-infected individuals were incubated with 50 μg/ml wildtype ESAT6-CFP10 or ROP-ESAT6-CFP10 for 18 hours (the third patient was from a different experiment). An individual cell producing IFN-γ is represented by appearance of a spot with number of spots at lower left corner. CD4 and CD8 counts are listed on the right of the cell picture. (**C**) ROP antigen does not stimulate T cell reaction in healthy donors.

Firstly, we incubated PBMC samples from eight clinically diagnosed TB patients with two ELISpot-based diagnostic kits. The commercially available T-SPOT.*TB* contains synthetically generated overlapping peptides from ESAT-6 (T-spot1) and CFP10 (T-spot2). In the second kit, the overlapping synthetic primers were replaced with an ROP based on two linked TB antigens (ESAT-6 and CFP10). Diagnosis of TB was found to be directly comparable between ROP-ESAT6-CFP10 and the established T-SPOT.*TB* assay in six out of the eight patients. In the remaining two samples, the kit based on ROP-ESAT-6-CFP 10 was more sensitive than the T-SPOT.*TB* assay (patients 1 and 6 - Figure 3A).

Next, to test the efficiency of ROP antigen presentation to CD8^+^ T cells, we used PBMCs from HIV and TB co-infected patients with depleted CD4^+^ lymphocyte levels and examined their ability to respond to TB antigens. PBMCs from patients were incubated with either ROP-ESAT6-CFP10, or a soluble wild-type form of the linked native antigen (ESAT6-CFP10). As shown in Figure 3B, IFN-γ production in response to the native antigen was dependent on the presence of CD4^+^ lymphocytes (Patient 2), suggesting epitopes could only be presented via MHC class II. In contrast, the ROP version of the same antigen was equally effective at inducing IFN-γ responses in CD4^+^ high (patient 2) and CD4^+^ depleted individuals (Patients 1, 3 and 4), suggesting that epitopes were presented efficiently to CD8^+^ T cells (Figure 3B). PBMCs isolated from healthy donors have no response or very low response to ESAT6-CFP10 or ROP-ESAT6-CFP10 stimulation (Figure 3C).

### ROP immunization protects mice from challenge with tumour cells expressing the cognate antigen

**Figure 4:**
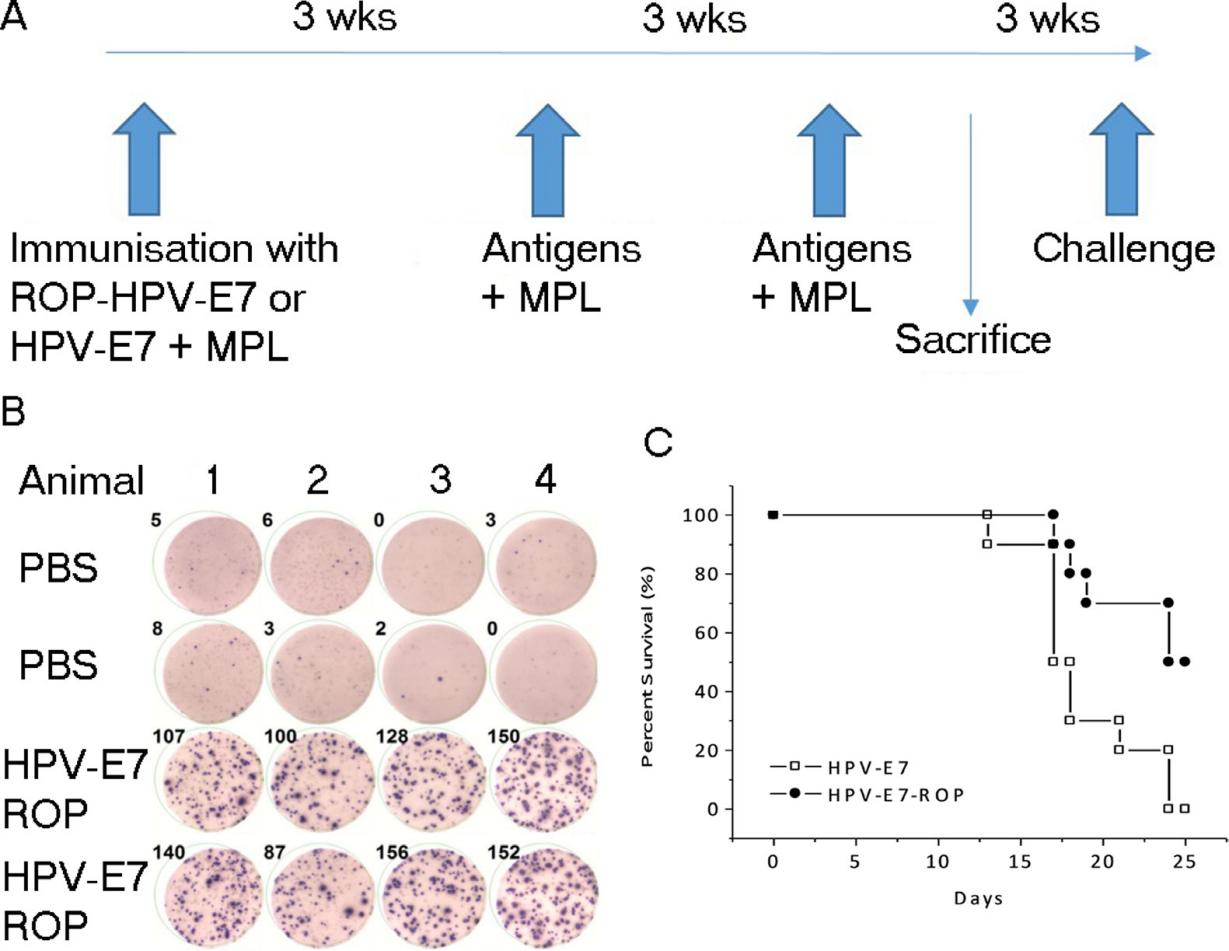
Immunization with ROP-HPV increases survival times in a mouse tumour model (**A**) Experimental timeline. Mice were immunized with ROP-HPV-E7 (*n* =14) or native HPV-E7 (*n* = 14) at day 0 and then further immunized twice at 3 week intervals with antigen ^+^ monophosphoryl lipid A (MPL). Four mice from each group were sacrificed for ELISpot analysis and the remaining mice were subjected to challenge with B16 cells expressing HPV-E7 3 weeks later. (**B**) IFN-γ release from activated lymphocytes. Following immunization PBMCs were incubated with an ELISpot test using ROP-HPV-E7 (data for native antigen primed mice not shown). (**C**) Survival rates for mice immunized with ROP-HPV-E7 compared to protein HPV-E7 following challenge with B16 cells expressing HPV-E7.

Human papilloma virus (HPV) and survivin are both associated with tumours. We next investigated the effect of ROP-HPV or ROP-survivin immunization on tumour models *in vivo*. Mice (14 per group) were immunized three times with either ROP-HPV-E7, native HPV-E7 or adjuvant alone with a three-week interval between immunizations. Three weeks after the last immunization, four mice from each group were sacrificed to assess antigen-specific responses. The remaining mice were challenged with B16 cells expressing HPV-E7 and survival rates were followed for 25 days after HPV expressed-B16 cells injection (Figure 4A). Immunization with both HPV-E7 protein and ROP-HPV-E7 resulted in PBMCs that were strongly reactive to re-challenge with the corresponding antigen (Figure 4B – only data for ROP-HPV-E7 shown); and significantly extended survival times. Furthermore, ROP-HPV-E7 was at least as effective as immunization with native HPV, with a strong trend towards enhanced survival (Figure 4C).

**Figure 5:**
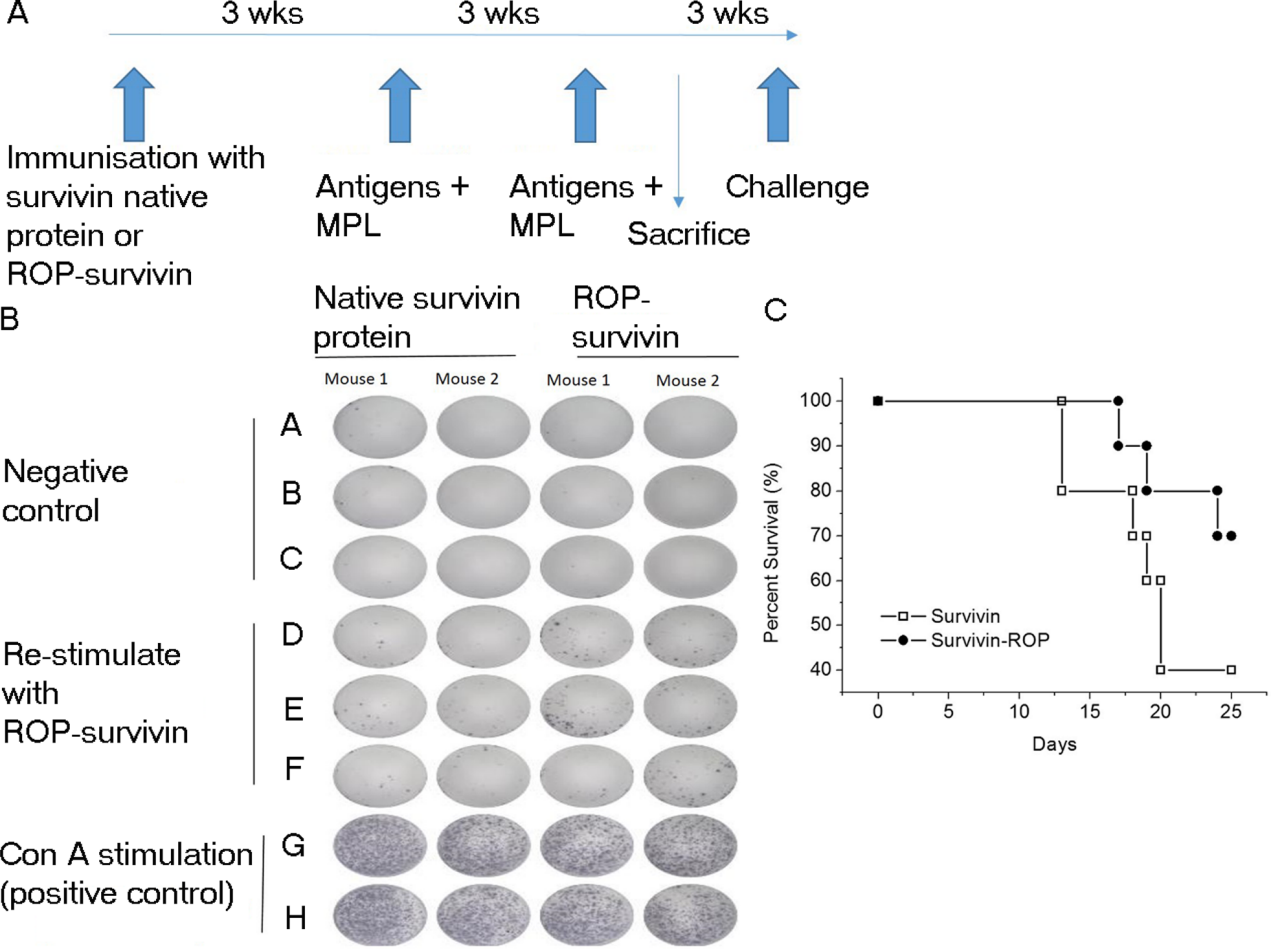
Immunization with ROP–survivin increases survival times in a mouse tumour model (**A**) Experimental timeline. Mice were immunized with ROP-survivin (*n* = 12) or native survivin protein (*n* = 12) at day 0 and then further immunized twice at 3 week intervals with antigen ^+^ MPL. Two mice from each group were sacrificed for ELISpot analysis and the remaining mice were subjected to challenge with B16 cells expressing survivin 3 weeks later. (**B**) IFN-γ release from activated lymphocytes. PBMCs were incubated with an ELISpot test using 50 *μ*g/ml ROP-survivin. (**C**) Survival rates for mice immunized with ROP-survivin compared to those immunized with native survivin protein following challenge with B16 cells expressing survivin.

A similar picture emerged when the experiment was repeated using ROP-survivin as the antigen (Figure 5A). Mice immunized with ROP-survivin or a vaccine based on native survivin protein both showed significant antigen specific responses, and these correlated with increased survival after challenge with B16 cells expressing survivin, though survival was significantly better in mice immunized with ROP-survivin (Figure 5B and 5C).

## DISCUSSION

Overlapping peptides have a compelling set of properties that make them very attractive candidates for vaccines designed to elicit T cell responses. They lack the viral or bacterial elements present in vector-based vaccines that can dominate the desired response or lead to damaging off target responses. Unlike single peptide epitope vaccines, they offer the potential for much broader responses, avoiding the need for HLA typing of individuals. They can also benefit from the improved persistence of response resulting from the use of longer peptides.

The use of ROPs as a more practical and economic way of generating sets of overlapping peptides brings their clinical application a significant step nearer. In this study, we have shown that *in vivo* processing of ROPs can occur if the peptides are connected by a suitable cleavage site. This suggests that initial loading with ROP leads to endosomal uptake into DCs, where the ROP is processed into overlapping peptides by cathepsin S. The peptides are then cross-presented on MHC class I molecules. This process has general applicability, as we have demonstrated that it occurs with ROPs based on four different antigens (ovalbumin, TB-CFP10-ESAT6, HPV-E7 and survivin).

Furthermore, the *in vivo* processed peptides are cross-presented efficiently, as measured by T cell effector function. This can occur even in CD4 lymphocyte depleted individuals. Finally, this functional *in vitro* presentation translates into effective *in vivo* immunity as shown by the ability to enhance survival when immunized mice are challenged with tumour cells carrying the cognate antigen.

Immunization with tumour specific antigens is an effective way of stimulating immune responses against tumours, but the magnitude and duration of the response is not usually sufficient on its own to result in tumour clearance. This has led to the conclusion that effective cancer immunotherapy will need to combine approaches that enhance immune responses - such as non-specific checkpoint inhibition and immune stimulation, with an antigen specific component involving immunization with tumour antigens [18, 19]. This study demonstrates that antigens based on recombinant overlapping peptides have significant potential as part of such a combination approach to immunotherapy.

## MATERIALS AND METHODS

### Molecular cloning

We designed synthetic cDNA genes encoding residues 242 to 352 of OVA, native ESAT6 and CFP10 of *Mycobacterium tuberculosis*, HPV-16 E7, survivin and the respective LRMK linked recombinant overlapping peptides (ROP-OVA, ROP-ESAT6-CFP10, ROP-HPV, ROP-survivin), with codon selection optimized for expression in *E. coli*. The genes were synthesized by the GeneArt DNA synthesis service. The synthesized cDNAs and the Bsa4 linearized vector pNIC28-Bsa4 (SGC Oxford) were treated with T4 DNA polymerase (30 min at 22°C) in the presence of 2.5 mM dCTP and dGTP respectively. T4 DNA polymerase was inactivated by incubation at 80°C for 20 min. T4 DNA treated PCR products and vector were mixed at a ratio of 1:50 for 10 min at 25°C. An aliquot of ligation products was used to transform DH5a competent cells. The positive clones were identified by colony PCR and the corresponding plasmids were used to transform *E.coli* BL21 (DE3) for protein expression.

### Protein expression and purification

A single transformed BL21 (DE3) colony was picked into LB (50 μg/ml Kan) and cultured overnight at 37°C. The overnight culture was then diluted 100 times with fresh LB and cultured until the OD600 reached 0.6. Then IPTG was added to final concentration of 0.5 mM. The cells were harvested at 16 hr post IPTG induction by centrifugation. The cell pellets were resuspended in lysis buffer (25 mM Tris-HCl, 200 mM NaCl, 2% Triton X-100, 10 mM imidazole, pH8.0) and were lysed by sonication. Insoluble fractions were separated by centrifugation at 20,000g for 45 min. For expressed soluble protein, Ni-NTA resin was then added to the soluble fractions for 30 min, followed by washing with 30 resin volumes of lysis buffer and eluted with lysis buffer containing 300 mM imidazole. For proteins forming inclusion bodies, the insoluble fractions were re-suspended in buffer (25 mM Tris-HCl, 200 mM NaCl, 8 M urea, 10 mM imidazole, pH8.0) and centrifuged at 20,000g for another 45 min, before applying to the Ni-NTA resin. Washing buffer and elution buffer also contained 8 M urea. For refolding, the eluted proteins were first buffer exchanged to 25 mM Tris-HCl, 200 mM NaCl, 0.5 arginine-HCl, pH 8.0 then to PBS using a PD10 column (GE Healthcare Life Sciences, UK).

### Maintenance of cell line

The dendritic cell line DC2.4 was a gift from K. Rock (University of Massachusetts, Worcester, Mass.) and was maintained in RPMI 1640 (Sigma, UK) supplemented with 10% heat-inactivated fetal calf serum (Sigma), 2 mM l-glutamine.

### Patients

TB and HIV-TB co-infected patients were recruited from the No.2 people's hospital of Dali. Samples and / or data obtained were collected with informed donor consent in full compliance with national and institutional ethical requirements (COREC number COREC06/Q1606/139). All study protocols and consent forms were approved by the institutional review boards of The No.2 People's Hospital of Dali.

### Mice and immunization

C57BL/10 mice were primed subcutaneously with 200 μg of ROP-HPV or ROP-survivin emulsified with 50 μl of monophosphoryl lipid A (MPL) (Sigma). Mice injected with MPL adjuvant plus HPV-E7 or survivin wild type protein were used as controls. They were boosted subcutaneously twice at 3-week intervals with the same vaccine emulsified with MPL. Three weeks after the last boost, 10 mice of each group were challenged with B16-E7 or B16-survivin, and 2 or 4 mice of each group were used for ELISpot assays.

### Isolation of human peripheral blood mononuclear cells (PBMC), B cells, dendritic cells, T cells and murine splenocytes

Heparin-treated human blood (10 ml) from patients was carefully added to lymphocyte-separation medium (density = 1.077) and centrifuged at 1500 g for 25 min. The PBMC layer was transferred to a new tube and washed twice with RPMI 1640 medium. The PBMCs were counted and 10^5^ cells/well were used for stimulation.

AntiCD19 Ab (Millipore, Watford, UK) was used to purify CD19^+^ B cells.

DCs were purified from human PBMCs. Briefly, adherent cells from PBMCs were cultured in the presence of 50 IU/ml IL-4 (PeproTech, Rocky Hill, US) and 1000 IU/ml GM-CSF (PeproTech, US) for 6 days.

CD19^+^ B cells were purified by attaching PBMCs to anti-CD19^+^ monoclonal antibody pre-coated plate. Cells were then re-suspended for the next step.

CD8^+^ T cells were purified by negative selection using microbeads kit (Miltenyi, Germany) as per the manufacturer's instructions.

Mouse spleens were meshed and loaded to lymphocyte separation medium Histopaque-1.083 (density = 1.083 g ml-1; Sigma), and centrifuged at 1500g for 25 min before transferring the layered lymphocytes to a new tube with cell culture medium. The cells were washed by RPMI 1640 and 1 × 10^5^ splenocytes/well were used for stimulation in ELISpot assays.

### ELISpot assays

Assays were performed using ELISpot kits (Mabtech, Sweden). Briefly, PBMCs or splenocytes were re-stimulated overnight with 10 μM ROP-ESAT6-CFP10, ROP-HPV, or ROP-survivin in anti-IFN-γ-Ab precoated plates (Millipore, Bedford, MA). Cells were discarded, and biotinylated anti-IFNγ antibodies were added for 2 hr at room temperature, followed by another 1hr of incubation at room temperature with anti-biotin antibody labeled with enzyme. After colour developed, the reaction was stopped by washing plates with tap water and plates were air-dried. Spots were counted with an Elispot reader (Autoimmun Diagnostike, Strasburg, Germany). Results were expressed as spot forming units/10^6^ cells.

### Antigen presentation assays

DC 2.4 cells were plated in 24 well plates (4 × 10^5^ per well) and incubated with the ROP-OVA or OVA proteins (both at 1 mg/ml in PBS) for 13 hr, and followed by flow cytometry analysis.

### Flow cytometry

At 13 hrs, cells were harvested by EDTA treatment and washed once using 1 ml ice-cold FACS buffer (2% FCS in PBS). PE conjugated monoclonal antibody 25.D1-16 was then added at a final concentration of 0.6 μg/ml. Cells were incubated for 30 min on ice in the dark and washed three times using 1 ml FACS buffer, re-suspended in 0.2 ml FACS fixing buffer (BD) and analyzed by flow cytometry using FlowJo software (ThreeStar, San Carlos, CA, USA).

### Statistical analysis

Statistical analysis was performed using the Student's *t* test. *P* values ≤ 0.05 were considered significant.

## Abbreviations

APC: Antigen presenting cells
CD19: Surface antigen found on B cells and follicular DCs, involved in up-regulating immune responses
CD4: Surface antigen found on lymphocytes involved in humoral responses
CD8: Surface antigen found on lymphocytes involved in cellular responses
CFP10-ESAT6: A 10 kDa antigen secreted by *Mycobacterium tuberculosis*
CTLA-4: Cytotoxic T cell antigen 4
DC: Dendritic cell
ER: Endoplasmic reticulum
H-2b: Major histocompatibility complex of the mouse
HPV: Human papilloma virus
MHC: Major histocompatibility complex
PBMC: Peripheral blood mononuclear cells
PD-1: Programmed cell death protein - 1
ROP: Recombinant overlapping peptide
ROP-HPV E7: ROP based on HPV E7 protein
ROP-Ova: ROP based on ovalbumin
ROP-survivin: ROP based on the tumour expressed protein survivin
TB: Tuberculosis
TCR: T cell receptor
